# Reconciliation of Decision-Making Heuristics Based on Decision Trees Topologies and Incomplete Fuzzy Probabilities Sets

**DOI:** 10.1371/journal.pone.0131590

**Published:** 2015-07-09

**Authors:** Karel Doubravsky, Mirko Dohnal

**Affiliations:** 1 Department of Informatics, Faculty of Business and Management, Brno University of Technology, Brno, Czech Republic; 2 Department of Economics, Faculty of Business and Management, Brno University of Technology, Brno, Czech Republic; Nankai University, CHINA

## Abstract

Complex decision making tasks of different natures, e.g. economics, safety engineering, ecology and biology, are based on vague, sparse, partially inconsistent and subjective knowledge. Moreover, decision making economists / engineers are usually not willing to invest too much time into study of complex formal theories. They require such decisions which can be (re)checked by human like common sense reasoning. One important problem related to realistic decision making tasks are incomplete data sets required by the chosen decision making algorithm. This paper presents a relatively simple algorithm how some missing III (input information items) can be generated using mainly decision tree topologies and integrated into incomplete data sets. The algorithm is based on an easy to understand heuristics, e.g. *a longer decision tree sub-path is less probable*. This heuristic can solve decision problems under total ignorance, i.e. the decision tree topology is the only information available. But in a practice, isolated information items e.g. some vaguely known probabilities (e.g. fuzzy probabilities) are usually available. It means that a realistic problem is analysed under partial ignorance. The proposed algorithm reconciles topology related heuristics and additional fuzzy sets using fuzzy linear programming. The case study, represented by a tree with six lotteries and one fuzzy probability, is presented in details.

## Introduction

There is a broad spectrum of decision-making tasks, e.g. engineering, economics, sociology, ecology, informatics etc., see e.g. [1–7]. These realistic decision-making tasks are often difficult to solve by limited available input information items (III).

Moreover, realistic industrial and natural science decision-making applications are based on vague data sets. For example, chemical, petrochemical and food processes take into decision making processes inaccurate measurements, e.g. gross systematics errors, chaotic changes of concentrations, flow rates, etc., see e.g. [8]. Bankruptcies, Loans and Investments are examples from economics. Management of many common resources, e.g. environment and safety problems, is implemented and solved via group interactions, which is best described by public goods games [9].

Most of decision-making tasks can be represented by single root trees, i.e. decision trees, and sets of available III, such as probabilities, penalties, plausibility, etc., see e.g. [8]. In real-world problems, the complete III set is usually not available; there is lack and uncertainty input information, see e.g. [10, 11].

If uncertainties cannot be quantified using a simple probabilistic way then the topic of possibility decision theory is often a natural one to consider, see e.g. [12, 13]. In many studies the problem of information uncertainty is handled using fuzzy sets, e.g. fuzzy numbers, see.e.g. [14–17].

Problems related to under-specification / lack information are usually solved by metaheuristics, see e.g. [18, 19]. If all information items, e.g. probabilities, penalties, required by a chosen decision making algorithm are not available then the decision task is solved under the total ignorance. It means that the decision tree topology is the only input information available. The traditional algorithms cannot take into consideration the individual heterogeneity / features and the topology of complex systems / networks [20, 21]. This is partially correct for simple structures as trees as well. EBAs (Entropy-based algorithms) can be applied under III conditions [22]. They cover a broad spectrum of task of different natures, see e.g. [23, 24].

If isolated information items are available then the decision task is solved under the so-called partial ignorance. Problems under partial ignorance are usually solved by Bayes' Theorem (Bayesian), see.e.g. [25, 26]. However well-established decision making algorithms, e.g. Bayesian [25, 27] cannot feasibly absorb these isolated additional information. This is particularly true if additional information items are represented by vague heuristics based on common sense reasoning.

Common sense heuristics are often the only available generators of III if the problem under total ignorance is solved. Such heuristic generates ARII (all required information items). However, the partial ignorance problem incorporates some AII (additional information items). ARII and AII are not consistent. A feasible reconciliation is inevitable. Common sense reasoning indicates that AII are more trustworthy. This fact must be taken into consideration.

Reconciliation algorithms are based on different formal tools, see.e.g. [28, 29]. There are several incomplete decision making problems and the relevant algorithms how to solve them [4, 6, 30–32].

Decision making algorithms will be accepted by users from industry, business etc. if they are simple. A decision making under total ignorance is simple if relevant heuristics are simple. The bellow proposed algorithm is a simple variant how to solve decision problems under partial ignorance. The advantage of this approach lies in the fact that is based on easy to understand heuristics.

## Heuristics

Decisions are often based on heuristics, see e.g. [33, 34]. This paper is based on an assumption that decision makers are ready to accept some general heuristics based on common sense reasoning. There are many possible heuristics, which can be mutually contradictory.

An example of a pair of mutually exclusive heuristics is

H1: A longer decision tree sub-path is less probable,
H2: A longer decision tree sub-path is more probable.

Both heuristics reflect some features of common sense reasoning. The heuristic H1 reflects, e.g. reliability and maintenance aspects [35]. A reliability interpretation of the heuristic H1 is:

Simpler Systems are More Reliable.

The heuristic H2 is usually much more specific. For example—If credit related tasks are studied then this heuristic becomes meaningful e.g.:

If a credit applicant satisfies more requirements then the final probability of the credit granting is higher.

This paper is based on the heuristics H1, however, it is a simple task to modify the below given theory using the heuristics H2.

**Fig 1:**
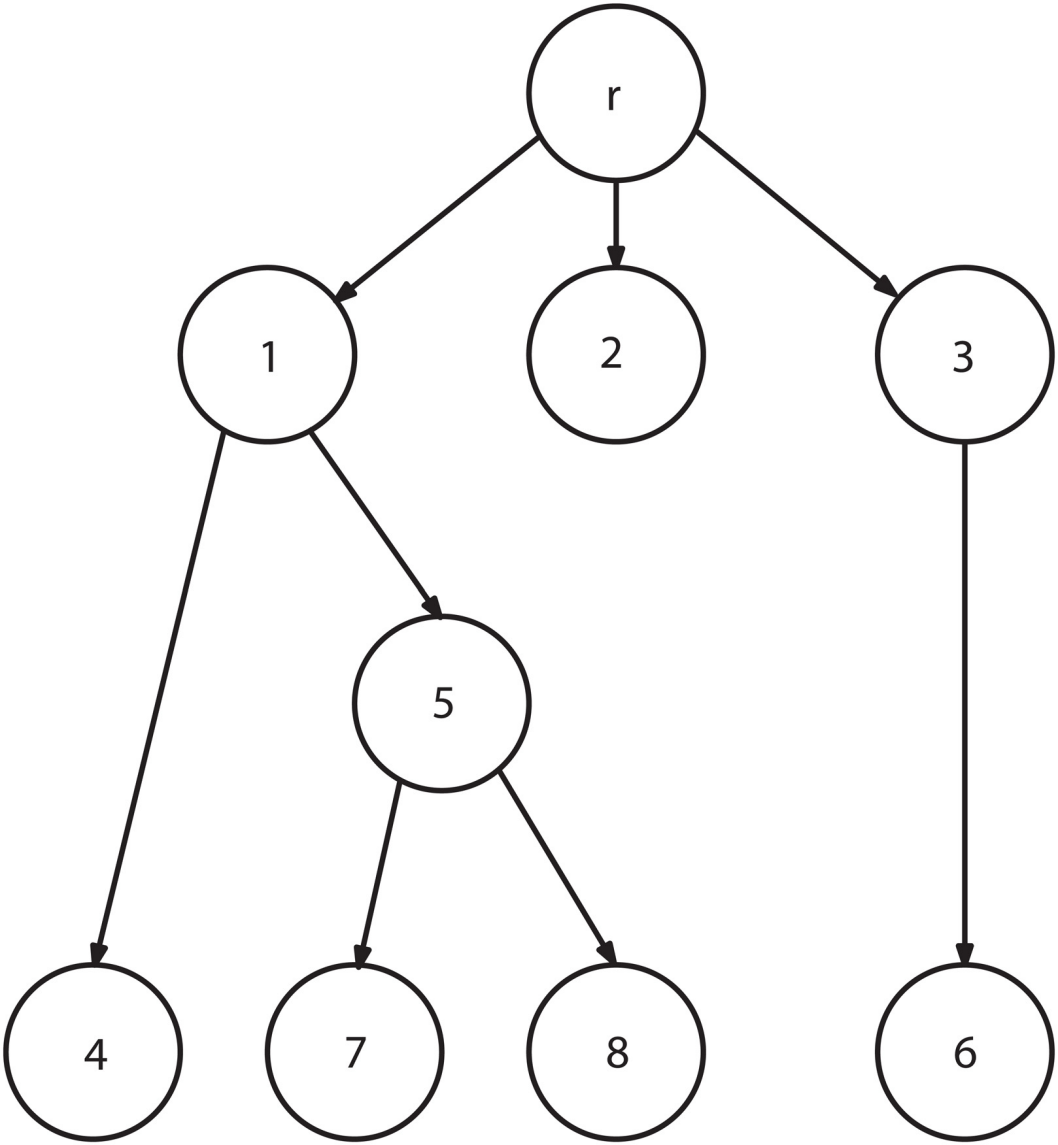
A decision tree.

The algorithm studied in this paper is based on a strong analogy between a water flow through a one root tree system of pipes and the decision tree of the same topology. Let us suppose that one litre of water is pumped into the root node of the decision tree each second and there is no accumulation of water inside the tree. The consequence is that one litre of water must leave the tree through its terminal nodes each second; see e.g. [36]. Flows through all branches of the tree under study must be balanced. The relevant balance equation for a node with *one* flow in, *k* flows out is written [14]:

$$IN_{i}=\sum_{j=1}^{k}OUT_{j}$$ (1)

where *IN* is flow into *i*th node and *OUT* are flows out of *i*th node. For example, the flow into node no. 1 is the sum of flows into nodes no. 4 and 5, see Fig 1. Flow balancing equations of a decision tree define a linear system of equations, see [14]:

$$A\cdot y^{T}=B$$ (2)

where *y* is a vector of flows.

This paper deals with the second case where the available information is not reliable and precise. There is a relationship between a flowrate through node and a probability of this node. Then a reinterpretation of the heuristic H1 is

- H3: The flowrate of water through a node is equal to its probability.

The following classical axiomatic definition of probability can be found in a number of standard texts [37]. Let A be an event, and Ω is a sample space. A probability *p(A)* of event *A*, must satisfy three axioms:

forall A∈Ω, p(A)≥0 (3)

p(Ω)=1 (4)

$$p(A_{1}\cup A_{2}\cup \ldots \cup A_{n})=\sum_{i=1}^{n}p(A_{i})$$ (5)

The relevant water flows through a node satisfies the axioms (3)–(5).

### Topological Resistance

A decision tree has one root node *r*, see e.g. Fig 1, where circles / nodes represent either lotteries or decisions [38].

The following definitions are used below:

*T* Set of terminals, see e.g. nodes 4, 6, 7 and 8, Fig 1.

*N* Set of all nodes.

*s*_*ij*_ Number of edges of the sub-tree where *i* is the root of the sub-tree and *j* is the node next to the sub-root, see e.g. *s*_*15*_ = 3 namely the edges 1–5, 5–7, 5–8.

*S*_*i*_ Number of all edges of the sub-tree where *i* is the sub-root (water resistance of *i*th node).

$$S_{i}=\underset{j}{\sum }s_{ij},\text{ see e.g. }S_{1}=s_{14}+\;s_{15}=\;1\;+\;3\;=\;4$$ (6)

where *j* represents nearest downstream node of the sub-tree next to the *i*th node.

s=ij*Si−sij, for all i, j∈N−T, s15*=S1–s15= 4 – 3 (7)

$S_{i}^{*}$ Number of edges of the *i*th sub-tree:

$$S_{i}^{*}=\underset{j}{\sum }s_{ij}^{*},\text{ see e.g. }S^{*}{}_{1}=s_{15}{}^{*}+s_{14}{}^{*}=\;4$$ (8)

where *j* represents nearest downstream node of the sub-tree next to the *i*th node.

To each node an appropriate splitting fraction is assigned [39]. This paper is based on the following definition of the splitting fraction *α*_*i*, *j*_. Splitting ratio from *i*th node to *j*th node:

αi,j=sij*Si*, for all j∈N−T, see e.g. α1,5=s15*/S*1= ¼ (9)

*p*_*j*_ of *j*th terminal for *j*∈*N* is a flowrate of water through *j*th node. The value *P*_*r*_ of a root node always equals one.

$$P_{r}=1$$ (10)

**Fig 2:**
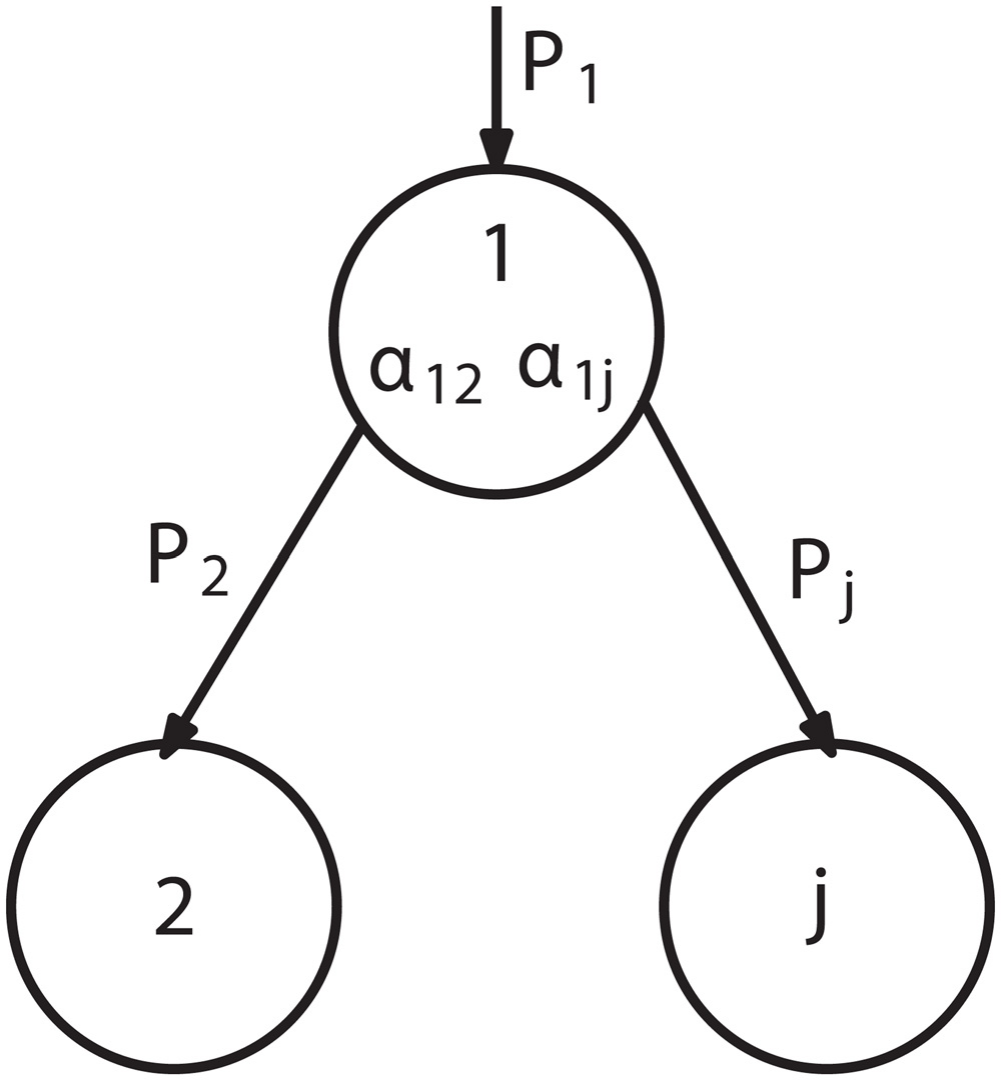
A flowrate of water through *j*th node.

Non-root node probability is, see Fig 2.

$$P_{j}=P_{i}\cdot \alpha_{i,j},\;j\in (N-T),$$ (11)

where *i* represents nearest upstream node (the sub-root node of the sub-tree). The Eq (11) is based on the balance Eq (1).

The set of *N*–*T* linear Eq (11) (the set of balance equations) where the set *P* is a vector of unknown variables and the splitting ratios *α* (9) are numerical constants can be easily solved.

*Example* The heuristic H3 can be used to evaluate resistances *S* (6), see Fig 1:

$$\begin{array}{lll} i\text{th node} & s_{ij} & S_{i}\\ \text{r} & s_{\text{r}1}=\;5,\;s_{\text{r}2}=\;1,\;s_{\text{r}3}=\;2 & 8\\ 1 & s_{14}=\;1,\;s_{15}=\;3 & 4\\ 2 & & -\\ 3 & s_{36}=\;1 & 1\\ 4 & & -\\ 5 & s_{57}=\;1,\;s_{58}=\;1 & 2\\ 6 & & -\\ 7 & & -\\ 8 & & - \end{array}$$

where symbol “-” in third column indicates the *i*th node is a terminal.

The splitting ratios (9) are:

αr,1=316=0.187αr,2=716=0.438αr,3=616=0.375αr,1+αr,2+αr,3=1α1,4=34=0.750α1,5=14=0.250α1,4+α1,5=1α3,6=1α5,7=12=0.500α5,8=12=0.500α5,7+α5,8=1 (12)

The system of linear Eq (11) with splitting ratios (12) is as follows, see Fig 1:

$$\begin{array}{l} 1=P_{r}\\ 0.187\cdot P_{r}=P_{1}\\ 0.438\cdot P_{r}=P_{2}\\ 0.375\cdot P_{r}=P_{3}\\ 0.750\cdot P_{1}=P_{4}\\ 0.250\cdot P_{1}=P_{5}\\ 1\cdot P_{3}=P_{6}\\ 0.500\cdot P_{5}=P_{7}\\ 0.500\cdot P_{5}=P_{8} \end{array}$$ (13)

Solving system of Eq (13) gives the following probabilities:

$$\begin{array}{l} P_{r}=1.000\\ P_{1}=0.187\\ P_{2}=0.438\\ P_{3}=0.375\\ P_{4}=0.140\\ P_{5}=0.047\\ P_{6}=0.375\\ P_{7}=0.024\\ P_{8}=0.024 \end{array}$$ (14)

## Partial Ignorance

A typical feature of all realistic decision tasks is a shortage of information. However, it is not true that nothing is known. The concept of the total ignorance represented by e.g. the heuristic H1 helps to incorporate a set of isolated specific information items within a general framework of the heuristics H3.

The set of additional information can be expresses by an additional set of probabilities for some nodes:

$$R\equiv \left(R_{1},R_{2},\ldots ,R_{h}\right)$$ (15)

has *h* elements, *h*≤*N*. For example the probability of node 2, see Fig 1 can be expressed by verbal value ‘*small*’:

$$R_{2}=small$$ (16)

The additional value(s) see Eqs (15) and (16) are reconciled with the heuristic H3. The first reconciliation step is to prove that the reconciliation is inevitable. The additional data set **R** Eq (15) is ignored, i.e. the total ignorance probabilities *P*_*TI*_ is solved. It means that the set of linear Eq (11) is solved and the set **P**_*TI*_ is quantified, see e.g. Eq (13). If

$$P_{TI}\;sufficiently\;equal\;R$$ (17)

then no reconciliation is required.

The concept sufficiently equal can be based on different soft calculus, e.g. fuzzy sets [40], rough sets. It is not studied in this paper.

### Fuzzy Reconciliation of Balancing Task

There are different types of reconciliation [41–43]. Data reconciliation consists in measured or estimated quantities (in our paper probabilities) in order to balance the flows in a given decision tree. The vector of flows *y* can be subdivided into vectors *x* and *u*, i.e. known probabilities *x* and totally unknown probabilities *u*, see [14]. Let us denote x* the vector of available probabilities. In general, the system *A*(*xu*)^*t*^ = *B* has no solution. The solved problem is to modify *x*, while remaining as close as possible to x*, such that the balance Eq (2), with *y* = (*xu*), are satisfied [14, 44]. There are several approaches to solve reconciliation problem.

The traditional, i.e. least-squares, approach to the reconciliation considers measured data sets. Measurement errors follow a normal distribution with zero average and a diagonal covariance matrix [14]. The accuracy of each measurement *x*_i_*, denoted as a mean value, is characterized by its standard deviation *σ*_*i*_. Data reconciliation becomes a problem of optimization under linear constraints. The solution of this problem is known by e.g. weighted least-squares, see [14, 45]. The main limitation of this approach is the fulfilment of the Gaussian hypothesis (a random variable must be unbounded). The other limitations are shown in [14].

Fuzzy interval reconciliation, see [14], is the approach based on estimated quantities (in our paper probabilities). Fuzzy interval reconciliation does not provide the same result as the least-square method. This approach provides only intervals instead of precise values. More or less possible values of each probability are limited by a fuzzy interval. Constraints of the mass balance Eq (2) are satisfied to a certain degree.

The problem of searching for a possible solution is based on a seeking an optimal position within all the (fuzzy) intervals of possible values, see [14]. This optimization problem can be solved by the max-min method, resolution methods such as α-cuts using fuzzy linear programming, see [8, 14, 46, 47]. The main limitation of this approach is the inability to detect a meaningful value of reconciliation (trade-off).

The reconciliation studied in this paper is based on fuzzy linear programming by [48] which provides a possibility to detect a meaningful value of reconciliation (trade-off). Its result is an optimal compromise:

Results of water probabilities (11) versus Additional probabilities (15).

The mathematical aspects of reconciliation are very important [49]. A fuzzy reconciliation is a solution of an over-specified set of equations:

A⋅P=B∪P=R (18)

where **A** is the matrix of the splitting ratios (9) and the equations **A·P** = **B** is given in (11), **P** = **R** is the set of additional probabilities.

The set of Eq (18) has *n + h* equations and *n* variables **P**. The reconciliation is solved by a fuzzy linear programming [50–54].

Eq (18) are differently important / reliable and therefore their violations are differently accepted by decision makers. Rather often certain equations cannot be violated at all. For example probability of a terminal event, see e. g. node 4 in Fig 1, is known very accurately. Therefore the corresponding probability P_4_ must be kept unchanged irrespective of any reconciliation algorithm.

**Fig 3:**
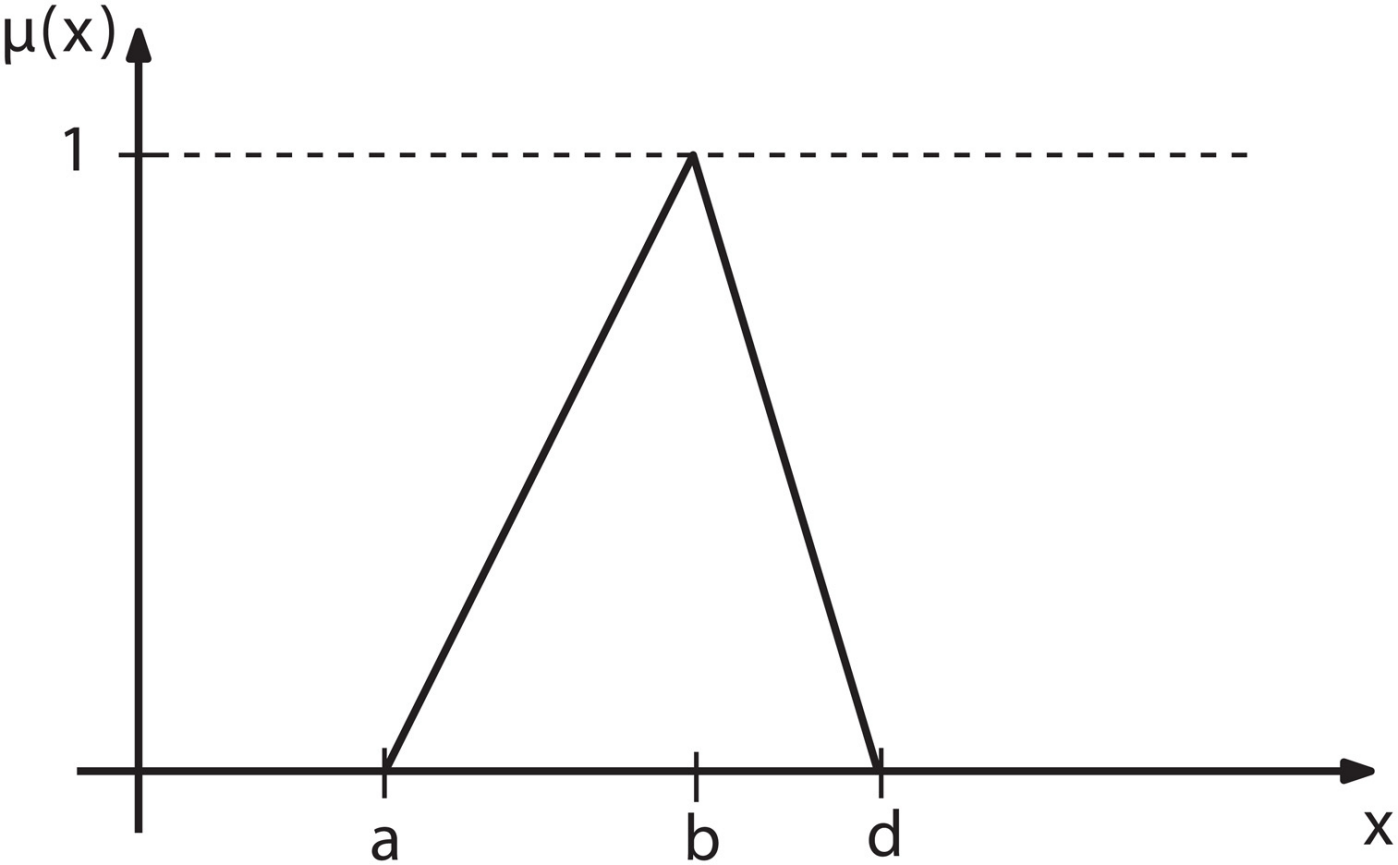
Triangular grades of membership.

Our experience indicates that the fuzzy set *R*_*i*_ is meaningfully characterized by triangular grades of membership [55], see e.g. Fig 3.

The triangular grades of membership can be expressed by a triplet (*a*, *b*, *d*), where

a<b<d (19)

Then the additional probability from (15) can be written as follows

$$P_{i}=R_{i}=\left(a,b,d\right)$$ (20)

The *i*-th Eq (20) can be transformed into four linear inequalities [47] by introducing two vectors of slack variables *S*_*u*_ and *S*_*l*_.

$$\sum_{i=1}^{h}\left(\alpha_{ij}\cdot P_{i}\right)+S_{uj}\geq b_{j}$$ (21)

$$\sum_{i=1}^{h}\left(\alpha_{ij}\cdot P_{i}\right)-S_{lj}\leq b_{j}$$ (22)

$$0\leq S_{uj}\leq b_{j}-a_{j}$$ (23)

$$0\leq S_{lj}\leq d_{j}-b_{j}$$ (24)

where

*b*_*j*_, *a*_*j*_, *d*_*j*_- see Eq (19),

*S*_*uj*_- is the *j*-th upper slack variable,

*S*_*lj*_- is the *j*-th lower slack variable.

The set of inequalities (21)–(24) represents fuzzy description of *j*-th linear Eq (11).

The total ignorance can generate rather misleading decision making results. Therefore it is highly desirable to over specify the problem by using additional information items (15). This set of additional information allows us to cross check not only the heuristics but the additional information items among themselves as well.

One possible objective function which represents a meaningful reconciliation (trade-off) is

Q=minSSul(∑j=1m(Suj/(bj−aj)+Slj/(dj−bj))) (25)

The minimization problem (25) can be finally solved as a conventional linear programming task [53].

For example the probability of node 2, see e.g. Fig 1, is defined as ‘*small*’ (16). This verbal value ‘*small*’ is expressed by a fuzzy set. The fuzzy set is characterized by its triangular grade of membership (19)

a=0.1; b=c=0.2; d=0.25 (26)

and can be written as *R*_2_ = (0.1;0.2;0.25). This equation can be transformed by (21)–(24) into four linear inequalities by introducing two slack variables *S*_*u*_ and *S*_*l*_.

$$\begin{array}{l} 0.1\cdot P_{2}+S_{u}\geq 0.2\\ 0.1\cdot P_{2}-S_{l}\leq 0.2\\ 0\leq S_{u}\leq 0.1\\ 0\leq S_{l}\leq 0.05 \end{array}$$ (27)

The inequalities (27) are added to the balance Eq (11). The objective function, see Eq (25) is

Q=minSSul(Su/0.1+Sl/0.05) (28)

The classical linear programming task (27), (28) is solved using available software e.g. GAMS [54].

The above described algorithm has the following advantages:

- It is simple and therefore transparent and easy to understand.
  Majority of well-established decision making and consequently reconciliation algorithms have not taken into consideration limits / extent of formal education (mathematics, computer science) of decision makers in e.g. industry, investment and public sector [56]. However, just simple and therefore transparent algorithms will be used extensively in industry, etc.
- The heuristics can be easily modified / changed to fit into common sense reasoning of decision makers.
  A set of potentially useful heuristics can be ad hoc generated for different branches of human activities, e.g. for safety studies—a complex system is less reliable. Such ad hoc heuristics can replace the general heuristics H1, H2 mentioned above.
- Complex decision trees can be easily treated.
  If the problems under complete ignorance are solved then the linear programming problem is the only limit. Therefore it is possible to solve very large trees.
- Incorporation of additional constraints is simple.
  Introduction of slack variables for each / some additional probability(ies) (20) allows us to have flexible control of the newly generated linear programming and perform, e.g. different sensitivity analyses.

## Illustrative Example

**Fig 4:**
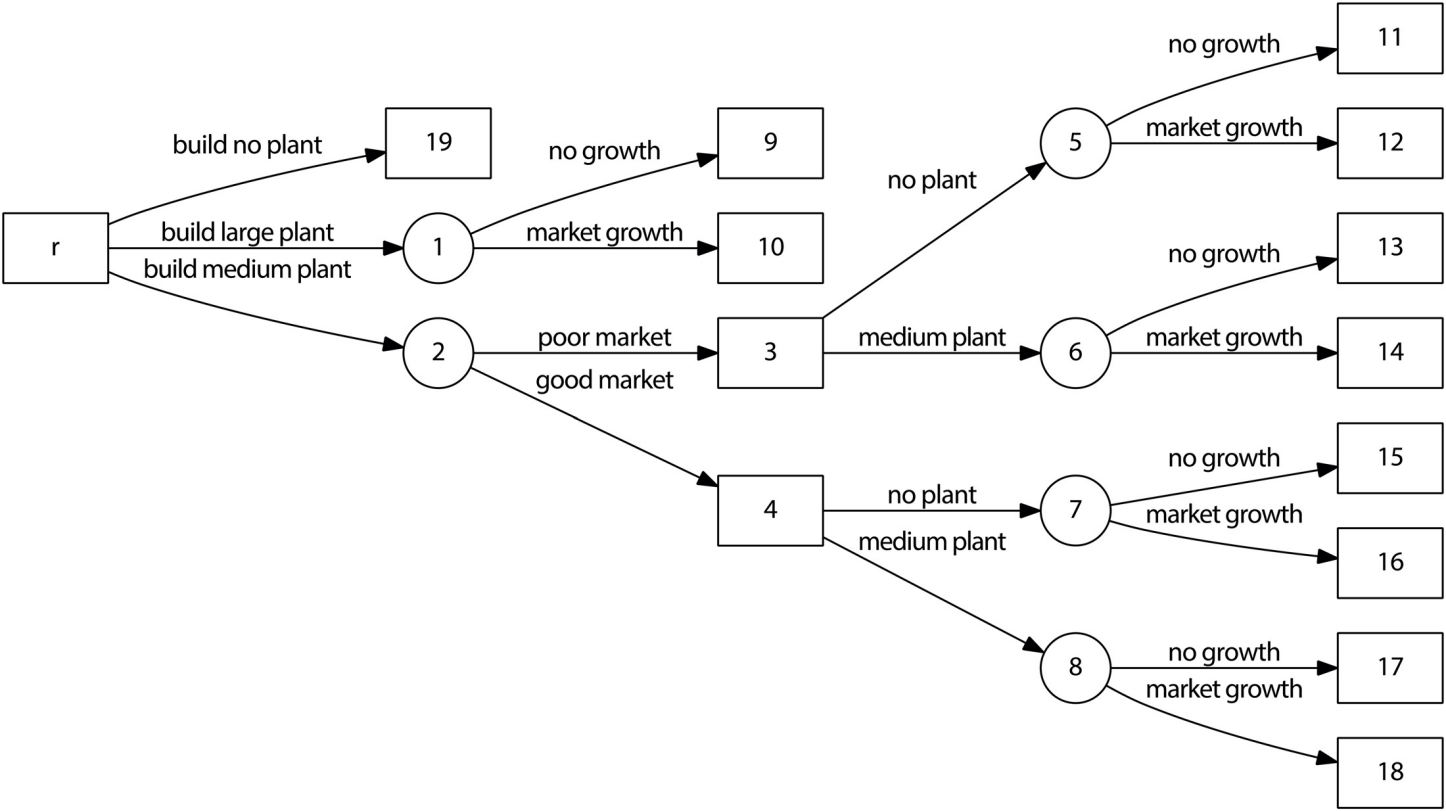
Decision Tree (Source: edited by [57]).

A modified version of the original decision tree [57], see Fig 4 and Table 1, is self-explanatory. The numbers of arcs given in Fig 4 are used to identify the corresponding probabilities. The decision tree under study contains many nodes and edges, where circles mean lottery nodes and squares mean decision nodes [57]. The tree has 20 nodes and 19 edges. The nodes 9–19 are terminal nodes. Additional probabilities **R** (15) are not given. It means that the total ignorance problem is solved, see Table 1.

**Table 1:** Profit and additional probabilities of terminal, see Fig 4.

Node	Probability	Profit (£)	Node	Probability	Profit (£)
**1**	-	-	**11**	-	–30,000
**2**	-	-	**12**	-	10,000
**3**	-	-	**13**	-	–70,000
**4**	-	-	**14**	-	–10,000
**5**	-	-	**15**	-	30,000
**6**	-	-	**16**	-	70,000
**7**	-	-	**17**	-	80,000
**8**	-	-	**18**	-	120,000
**9**	-	20,000	**19**	-	0
**10**	-	100,000			

The heuristic H1 is used to assess the probabilities. The resulting splitting ratios (9) are given in Table 2.

**Table 2:** Splitting ratios for all non-terminal nodes, see Fig 4.

Node	Splitting ratios	Node	Splitting ratios
**r**	*α*_*r*,1_ = 0.4211, *α*_*r*,2_ = 0.1052, *α*_*r*,19_ = 0.4736	**5**	*α*_5,11_ = 0.5, *α*_5,12_ = 0.5
**1**	*α*_1,9_ = 0.5, *α*_1,10_ = 0.5	**6**	*α*_6,13_ = 0.5, *α*_6,14_ = 0.5,
**2**	*α*_2,3_ = 0.5, *α*_2,4_ = 0.5	**7**	*α*_7,15_ = 0.5, *α*_7,16_ = 0.5,
**3**	*α*_3,5_ = 0.5, *α*_3,6_ = 0.5	**8**	*α*_8,17_ = 0.5, *α*_8,18_ = 0.5,
**4**	*α*_4,7_ = 0.5, *α*_4,8_ = 0.5		

The resulting probabilities are given in Table 3.

**Table 3:** Node probabilities.

Node	Probability	Node	Probability
**r**	1	10	0.2106
**1**	0.4211	11	0.01315
**2**	0.1052	12	0.01315
**3**	0.0526	13	0.01315
**4**	0.0526	14	0.01315
**5**	0.0263	15	0.01315
**6**	0.0263	16	0.01315
**7**	0.0263	17	0.01315
**8**	0.0263	18	0.01315
**9**	0.2106	19	0.4736

The problem under partial ignorance is represented by the total ignorance problem; see Table 1 and Fig 4. and one additional probability R. The only additional probability R is the probability of the *Good Market*, see Fig 4. The above described procedure (18)–(25) is used to solve three cases of *Good Market* probabilities, see Table 4.

**Table 4:** Fuzzy Probabilities I, II, III for *Good Market (R*_*4*_ *)*.

	a	b = c	D
**I**	0.05	0.07	0.08
**II**	0.04	0.09	0.10
**III**	0.11	0.16	0.17

There are 20 nodes in Fig 4, therefore 20 variables (*P*_*r*_–*P*_*19*_) are used and 20 linear Eq (9) are compiled. The number of additional probabilities is 1. Therefore the total number of equations is 20 + 1 = 21. The first fuzzy probability I, see Table 4, gives the following equation:

$$P_{4}=R_{4}=\left(0.05;0.07;0.08\right)$$

This equation is transformed, using Eqs (21)–(24), into the following four linear inequalities by introducing four slack variables *S*_*u1*,_ *S*_*l1*_.

$$P_{4}+S_{u1}\geq 0.07$$

$$P_{4}-S_{l1}\leq 0.07$$

$$0\leq S_{u1}\leq 0.02$$

$$0\leq S_{l1}\leq 0.01$$

The system of 24 constraints (equations) is solved together with the objective function (25) using a linear programming algorithm. Table 5 gives the resulting probabilities based on the splitting ratios (9), balance Eq (11) and partial ignorance value for each fuzzy probability from Table 4.

**Table 5:** Fuzzy approach—calculated probabilities.

Node	r	1	2	3	4	5	6	7	8	9	10	11	12	13	14	15	16	17	18	19
**Probab. (I)**	1	0.4211	0.1052	0.0352	0.07	0.0176	0.0176	0.035	0.035	0.2106	0.2106	0.0088	0.0088	0.0088	0.0088	0.0175	0.0175	0.0175	0.0175	0.4736
**Probab. (II)**	1	0.4211	0.1052	0.05	0.055	0.025	0.025	0.0276	0.0276	0.2106	0.2106	0.0125	0.0125	0.0125	0.0125	0.0138	0.0138	0.0138	0.0138	0.4736
**Probab. (III)**	No solution																			

The details of the calculations are listed in the Table 6.

**Table 6:** Violation details.

	Equation	*S_u_*	*S_l_*	Value of membership function	Value of objective function Q, see Eq (25)
**I**	21 (*P*_*4*_)	0.00	0.00	1.00	0.00
**II**	21 (*P*_*4*_)	0.035	0.00	0.30	0.70
**III**	21 (*P*_*4*_)	The corresponding linear programming task has *No Solution*			

The deviations S_u_ and S_l_ are zero for the first case I. It means that the corresponding equations (21)–(24) are satisfied perfectly. In the second case II, the corresponding equations are not satisfied perfectly. It means that it is a compromise solution (with value 0.7, see Table 6).

The evaluated probabilities, see Table 5, allow us to solve the decision making problem using some classical methods, e.g. the expected value criterion [57].

The first case I (Table 4) the expected value of profit (the expected value criterion) is 63 150 £. The company should build the medium plant in the first step. If the market is good then the company should build the other medium plant. If the market is poor then the company should build no plant.

The second case II (Table 4) the expected value of profit is 60 000 £. The company should build the large plant in the first step.

Table 7 gives the resulting probabilities based on the least-squares method for each initial probability, i.e. 0.07, 0.09 and 0.16, from Table 4. All calculations, see Table 7, were performed using GNU Octave, see e.g. [58].

**Table 7:** Least-squares approach—calculated probabilities.

Node	r	1	2	3	4	5	6	7	8	9	10	11	12	13	14	15	16	17	18	19
**Probab. (I)**	1	0.420	0.0875	0.0293	0.058	0.0147	0.0147	0.0291	0.0291	0.2102	0.2102	0.0098	0.0098	0.0098	0.0098	0.0170	0.0170	0.0170	0.0170	0.4727
**Probab. (II)**	1	0.421	0.0976	0.0463	0.051	0.0232	0.0232	0.0256	0.0256	0.2104	0.2104	0.0127	0.0127	0.0127	0.0127	0.0139	0.0139	0.0139	0.0139	0.4732
**Probab. (III)**	No solution																			

Tables 5 and 7 show that the difference between the additional probabilities (15), see Table 4, and the resulting probabilities (11) is smaller if the fuzzy linear programming is used than in the case of the least-squares method.

## Conclusion

Evaluations of realistic complex decision trees attract attention. Decision makers / field experts in their desperation to satisfy increasingly demanding laws and regulations (e.g. safety and environmental engineering) and / or pressure of competition (e.g. exchange rates hedging) are ready to believe that there is a theoretical answer to their needs. However, the only solution is to increase data / knowledge inputs into decision making processes. It means that no available information item may be ignored. Therefore known isolated fuzzy probabilities must be meaningfully incorporated into the decision making tasks.

The key reconciliation problem is the choice of the probabilities generation heuristic. If this heuristic is not accepted by a decision maker then some modifications of this heuristic are inevitable to cover specific requirements of the decision making problem under study. This is, however, an ad hoc procedure.

The heuristic used in this paper is based on a strong analogy between a water flow through a one root tree system of pipes and the decision tree of the same topology. The heuristic solves decision problems under total ignorance, i.e. the decision tree topology is the only information available. However, isolated information items e.g. some vaguely known probabilities (e.g. fuzzy probabilities) are usually available. It means that a realistic problem is analysed under partial ignorance. The present paper shows how a fuzzy linear programming is used to reconcile the probabilities generated by the water heuristic and the available set of fuzzy probabilities.

The presented algorithm is a simple variant how to solve decision problems under partial ignorance. The advantage of this approach lies in the fact that the objective function and the equations of the individual constrain are linear, and therefore easily solvable using commonly known simplex method. However, it is an easy task to incorporate not just fuzzy probabilities but e.g. fuzzy penalties and profits into trees evaluations / reconciliations. In mathematical terms it means to use more complex fuzzy optimization algorithms [17, 48]. Moreover different heuristics can be used to generate missing values. The negative consequence is that multidimensional nonlinear optimization problems must be solved.

Decision trees can be generated using different algorithms. For example a genetic algorithm can induce decision trees [59]. It means that not just missing values but some trees themselves can be generated.

Limitation of proposed approach is the need to build decision tree (it can be large for extensive projects). Also it should be noted that the proposed heuristics H1 is not the only one possible and it may not suit all decision-making problems. The choice of suitable heuristics depends on the type of solved decision-making problem. It means it depends on the decision maker requirements. Water heuristic H1 can be replaced by more general entropy based heuristics using e.g. powers degrees [60].

The proposed approach have broad spectrum of applications, e.g. investment, biotechnology, chemical engineering and medicine. Moreover it is possible to take into consideration tasks where the probabilities are given vaguely, e.g. using fuzzy numbers, or they are specified from different sources, e.g. from different members of project teams, experts of project management, etc.
